# Gene introduction into the mitochondria of *Arabidopsis thaliana* via peptide-based carriers

**DOI:** 10.1038/srep07751

**Published:** 2015-01-13

**Authors:** Jo-Ann Chuah, Takeshi Yoshizumi, Yutaka Kodama, Keiji Numata

**Affiliations:** 1 Enzyme Research Team, Biomass Engineering Program Cooperation Division, Center for Sustainable Resource Science, RIKEN, 2-1 Hirosawa, Wako-shi, Saitama 351-0198, Japan; 2 Institute for Advanced Biosciences, Keio University, 403-1 Nipponkoku, Daihoji, Tsuruoka, Yamagata 997-0017, Japan; 3 Center for Bioscience Research and Education, Utsunomiya University, Tochigi 321-8505, Japan

## Abstract

Available methods in plant genetic transformation are nuclear and plastid transformations because similar procedures have not yet been established for the mitochondria. The double membrane and small size of the organelle, in addition to its large population in cells, are major obstacles in mitochondrial transfection. Here we report the intracellular delivery of exogenous DNA localized to the mitochondria of *Arabidopsis thaliana* using a combination of mitochondria-targeting peptide and cell-penetrating peptide. Low concentrations of peptides were sufficient to deliver DNA into the mitochondria and expression of imported DNA reached detectable levels within a short incubation period (12 h). We found that electrostatic interaction with the cell membrane is not a critical factor for complex internalization, instead, improved intracellular penetration of mitochondria-targeted complexes significantly enhanced gene transfer efficiency. Our results delineate a simple and effective peptide-based method, as a starting point for the development of more sophisticated plant mitochondrial transfection strategies.

In plants, the mitochondrion plays the fundamental role of energy generation and is involved in the detection of developmental signals, biotic and abiotic stresses^1^. The organelle also functions in reactive oxygen species induction and signaling, genome maintenance, respiration, and programmed cell death^2^. Methods that enable access into the plant mitochondria thus offer exciting avenues for study and manipulation of these essential physiological and biochemical processes. The mitochondrion is also a prospective new target for genetic engineering of plants, as an alternative to modification of the nucleus or plastids. Simultaneous expression of transgenes in different organelles may occur via the transformed mitochondria through interorganellar communication pathways within the cell^3^,^4^, as an effective approach to metabolically engineer the plant cell factory. Plant cells have shown great potential as hosts for the production of valuable medicinal compounds, pharmaceutically important secondary metabolites, recombinant proteins, flavors, fragrances, and colorants – none of which can be produced by microbial cells or chemical synthesis^5^.

Although the ability to introduce exogenous genes into plant mitochondria has wide biotechnological and fundamental significance, this complex organelle could not be transformed in whole plants with currently existing methodologies^6^. The double membrane and small size of the organelle are major obstacles in mitochondrial transfection^7^. To date, mitochondrial transformation has only been achieved using isolated plant mitochondria (by electrotransformation^8^ or natural competence^9^), as well as in yeast^10^,^11^ and *Chlamydomonas*^12^,^13^ (both by biolistic method), however this milestone has not yet been accomplished for any vascular plant. Mitochondrial transformation by microparticle bombardment^10^,^11^,^12^,^13^ is disadvantageous in terms of low frequency of transformant recovery. The lack of selectable markers for mitochondrial transformation adds to the challenge.

Peptides are a highly versatile and efficient class of transporters with the ability to translocate the cellular membrane and/or organellar membranes, and their potential use as gene delivery vectors has been substantiated in numerous studies^14^,^15^,^16^,^17^,^18^,^19^,^20^. Taking advantage of their innate nature, we aimed to develop a peptide-based carrier for delivery of genes specifically to the mitochondria in plants, where the peptide sequences were rationally determined based on the vast range of available information from associated studies in the past.

## Results

### Delivering plasmid DNA using the designed mitochondria-targeting peptide

**Figure 1:**
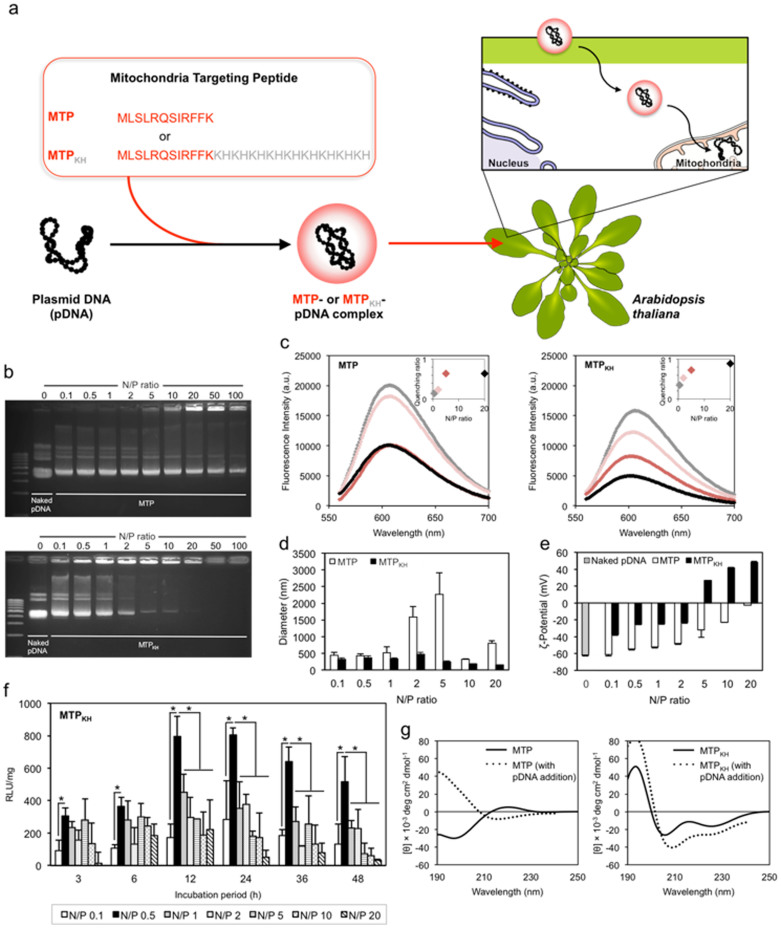
Mitochondria-targeting peptide as vector for delivery of pDNA into the leaves of *A. thaliana* localized to the mitochondria. (a) Schematic representation of the gene delivery strategy using MTP or MTP_KH_. (b) Electrophoretic mobility of pDNA in MTP- or MTP_KH_-pDNA complexes. (c) Condensation of pDNA by MTP or MTP_KH_ monitored by the EtBr exclusion assay. The inset shows the quenching ratio as a function of N/P ratio. (d,e) Hydrodynamic size and ζ-potential of MTP- or MTP_KH_-pDNA complexes measured by dynamic light scattering (DLS). Error bars represent standard deviations (*n* = 3). (f) Efficiency of pDNA delivery by MTP_KH_ determined using RLuc assay. Asterisks (*) indicate significant differences (Tukey's HSD test; *P* < 0.05). Error bars represent standard deviations (*n* = 4). (g) CD spectra of MTP or MTP_KH_ and respective complexes with pDNA in 40% TFE.

The mitochondria-targeting component consists of the first 12 amino acids of the presequence belonging to the yeast cytochrome *c* oxidase subunit IV that was capable of directing attached mouse cytosolic dihydrofolate reductase into the yeast mitochondrial matrix, both in vitro and in vivo^21^. The mitochondria-targeting dodecapeptide (hereafter referred to as MTP) was fused to a polycationic copolymer of alternating histidine and lysine residues (Fig. 1a). Polycations comprising histidine and lysine residues have proven to be beneficial in increasing cell transfection efficiencies^16^,^17^,^20^ and should, as we anticipate, help to facilitate electrostatic complexation of the resultant fusion peptide (designated MTP_KH_) and the polyanionic plasmid DNA (pDNA).

The stability of complexes formed upon binding of MTP and MTP_KH_, individually, with pDNA was investigated by an electrophoretic mobility shift assay (Fig. 1b). MTP exhibited weak pDNA binding and caused only a slight retention in pDNA shift even at a high N/P (defined as the number of amine groups from the peptide/the number of phosphate groups from pDNA) ratio of 100. The addition of polycations to MTP, particularly at higher concentrations (N/P 2 and above), proved to be essential in stabilizing the formed complexes. MTP_KH_ severely impaired pDNA mobility at N/P 2 and complex stability continued to increase with N/P ratio until complete retardation of pDNA movement was attained at N/P 20.

We also examined the tendencies of MTP and MTP_KH_ to condense pDNA by an ethidium bromide (EtBr) exclusion assay, as pDNA condensation is required for receptor-mediated uptake of complexes^22^. The binding of peptide causes conformational changes to the pDNA, resulting in displacement of intercalated EtBr which subsequently quenches the fluorescence from the EtBr-pDNA complex. At each of the distinct N/P ratios (0.5, 2, 5, 20) used for complex preparation in this assay, MTP_KH_ quenched the fluorescence with better efficiency than MTP, and achieved nearly complete quenching (approximately 90%) at N/P 20 (Fig. 1c).

The size of particles formed using MTP were relatively large and independent of polycation concentration (Fig. 1d), coherent with its poor pDNA binding/compacting abilities. In contrast, MTP_KH_ enabled formation of more compact particles with hydrodynamic diameters averaging between 140 nm and 480 nm, which are compatible with the caveolae-mediated endocytic pathway^23^,^24^. The surface charges of particles, which can contribute to cellular uptake^15^,^23^,^25^, were subsequently characterized (Fig. 1e). The ζ-potential of MTP-pDNA complexes were negative at all N/P ratios, although decrease in negative charges were seen with increasing N/P ratio, whereas those of complexes formed using MTP_KH_ transitioned from negative (N/P 0.1 to 2) to positive (N/P 5 to 20).

Next, we proceeded to evaluate the functionality of the designed MTP_KH_ in mitochondria-targeted gene delivery. MTP_KH_-pDNA complexes prepared at N/P ratios ranging from 0.1 to 20, as before, were infiltrated into the leaves of *Arabidopsis thaliana*, which serves as a model plant system. The pDNA harbors the gene encoding *Renilla* luciferase (RLuc) enzyme, which is expressed under the control of the mitochondrial-specific cyclooxygenase-2 (COX-2) promoter^26^. Expression of the reporter gene in the mitochondrial compartment of leaves, sampled at intermittent time points, was quantitatively determined by an RLuc assay. Complexes prepared at N/P 0.5 demonstrated remarkable transfection ability, well surpassing the transfection levels mediated by complexes of the other N/P ratios, and this interesting observation was true for all incubation periods tested (Fig. 1f). In contrast, complexes formed using MTP or lysine-histidine copolymer (N/P 0.5) or naked pDNA, when delivered to leaves, mediated negligible levels of expression (Supplementary Fig. 1).

Secondary structures of MTP and MTP_KH_, as well as their pDNA-bound versions (representative N/P ratio of 0.5, the most transfection-effective formulation), were therefore analysed by circular dichroism (CD) (Fig. 1g). MTP and MTP-pDNA showed either a random or low level of structuring. In contrast, the spectra of MTP_KH_ exhibited characteristic features of an α-helical structure (double minima at 207 and 222 nm)^27^ and the α-helicity was retained even upon complexation with pDNA.

### Enhancing pDNA delivery by addition of a cell-penetrating peptide

**Figure 2:**
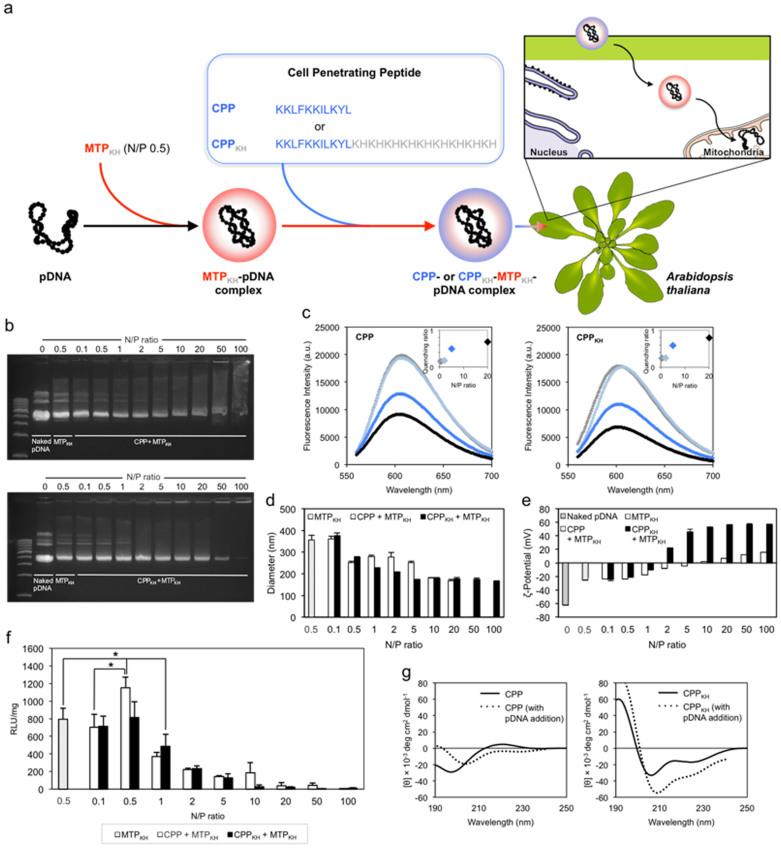
Combination of mitochondria-targeting peptide and cell-penetrating peptide as vector for delivery of pDNA into the leaves of *A. thaliana* localized to the mitochondria. (a) Schematic representation of the gene delivery strategy using CPP-MTP_KH_ or CPP_KH_-MTP_KH_. (b) Electrophoretic mobility of pDNA in CPP- or CPP_KH_-MTP_KH_-pDNA complexes. (c) Condensation of pDNA by CPP or CPP_KH_ monitored by the EtBr exclusion assay. The inset shows the quenching ratio as a function of N/P ratio. (d,e) Hydrodynamic size and ζ-potential of CPP- or CPP_KH_-MTP_KH_-pDNA complexes measured by DLS. Error bars represent standard deviations (*n* = 3). (f) Efficiency of pDNA delivery by CPP-MTP_KH_ or CPP_KH_-MTP_KH_ determined using RLuc assay. Asterisks (*) indicate significant differences (Tukey's HSD test; *P* < 0.05). Error bars represent standard deviations (*n* = 4). (g) CD spectra of CPP or CPP_KH_ and respective complexes with pDNA in 40% TFE.

The net negative surface charge of the MTP_KH_-pDNA complex (N/P 0.5) was fortuitous as it inspired a strategy, which we discovered later, to significantly improve the performance of our designed pDNA carrier. By the same principle that enabled interaction between the negatively-charged pDNA molecule and positively-charged MTP_KH_, we allowed new cell-penetrating components to electrostatically interact with negatively-charged MTP_KH_-pDNA complexes at N/P 0.5. The cell-penetrating peptides used were: (i) the BP100 peptide (hereafter referred to as CPP) and (ii) the fusion of CPP to the same copolymer of alternating lysine and histidine residues as before (designated CPP_KH_) (Fig. 2a). Both CPP and CPP_KH_ have displayed potential as efficient plant cell-penetrating tools^14^,^17^.

The resultant CPP- or CPP_KH_-MTP_KH_-pDNA complexes were characterized biophysically and functionally, as done previously. Addition of CPP and CPP_KH_ further stabilized the MTP_KH_-pDNA complexes and a gradual increment in stability was seen with increasing N/P ratio until complete charge neutralization occurred at N/P 100 for both peptides (Fig. 2b). CPP and CPP_KH_ also exhibited similar tendencies for pDNA condensation at each of the different N/P ratios (Fig. 2c). No apparent differences in average diameters were detected between complexes formed using CPP and CPP_KH_ at each N/P ratio, except when CPP was added at higher N/P ratios of 50 and 100, highly polydisperse particles were formed (Supplementary Table 1). Particle sizes were in the narrow range of 160–280 nm (Fig. 2d) and in terms of surface charge, ζ-potential values gradually transitioned from negative to positive for both CPP- and CPP_KH_-based complexes (Fig. 2e).

The various formulations were used to transfect *A. thaliana* leaves, in a similar manner as with the MTP_KH_-pDNA complexes, maintaining the 12 h incubation period that was previously determined to be sufficient for mitochondrial expression of the RLuc reporter gene. Results from evaluation by RLuc assay showed a marked increase in transfection levels, more than that of MTP_KH_-pDNA complexes, only by supplementation of CPP at N/P 0.5 (Fig. 2f). These transfection-optimized complexes, formed by sequential mixing of MTP_KH_ and CPP (both at N/P 0.5) with pDNA, displayed a relatively uniform distribution in size when observed by scanning electron microscopy (SEM) (Supplementary Fig. 2). Through additional experiments, we found no/low levels of extramitochondrial RLuc gene expression (i.e. cytosolic expression) by the mitochondrial-specific COX-2 promoter (Supplementary Fig. 3). We also performed conformational analysis by CD and found that both CPP and CPP_KH_ were α-helical when bound to pDNA (Fig. 2g).

**Figure 3:**
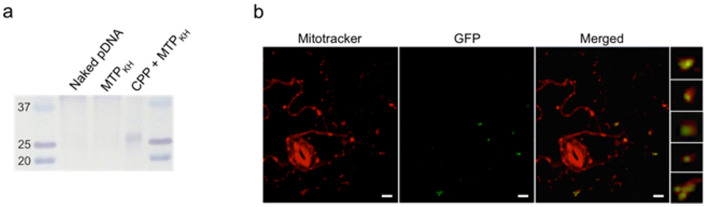
GFP expression in the mitochondria of *A. thaliana* leaf 12 h after transfection. (a) Western blot analysis of crude mitochondrial extracts from leaves previously infiltrated with naked pDNA, or complexes of MTP_KH_ (N/P 0.5) or CPP-MTP_KH_ (N/P 0.5 for each peptide) with pDNA. Data is representative of two independent experiments. (b) Confocal laser scanning microscope observation of epidermal cells of a leaf previously infiltrated with CPP-MTP_KH_-pDNA complexes (N/P 0.5 for each peptide). Enlarged images of several mitochondria with GFP expression are shown in the extreme right panel. Scale bars indicate 10 *μ*m.

As a final step, the efficiency of our designed peptide-based carrier was verified by delivery of pDNA containing a gene that encodes another well-known reporter, the green fluorescent protein (GFP), also under the control of the same mitochondrial COX-2 promoter. For comparison, leaves were infiltrated with either naked pDNA, MTP_KH_-pDNA or CPP-MTP_KH_-pDNA, and mitochondrial expression of GFP was subsequently detected by Western blotting using an anti-GFP antibody. Of the three different formulations, only the CPP-MTP_KH_ combination mediated pDNA delivery and significant levels of GFP expression, as evident from the band corresponding to the size of 27 kDa GFP (Fig. 3a). Confocal laser scanning microscopy provided visual confirmation of GFP expression, localized in the mitochondria of epidermal cells of leaves transfected using the optimized peptide-pDNA formulation (MTP_KH_ and CPP at N/P 0.5 each) (Fig. 3b).

## Discussion

We sought to understand the merits of the MTP_KH_-pDNA complex (N/P 0.5) over other formulations in enabling efficient gene transfer by correlating the physicochemical properties of complexes and their corresponding transfection efficiencies. Clearly, an optimal balance was achieved with the formulation (MTP_KH_-pDNA at N/P 0.5) in terms of binding strength. MTP_KH_ could interact with pDNA in vitro and subsequently dissociate to release the cargo in vivo, where the interaction, at the same time, compacted pDNA into a form ideal for its uptake in cells (toroid/doughnut structure proposed in these studies^22^,^28^). Although MTP_KH_, at N/P ratios higher than 0.5, resulted in increasingly stable formulations, a previous report showed that peptides with weak affinity for pDNA favour toroidal compaction^29^. The ability of peptides to aggregate with pDNA, is in fact, highly dependent on peptide structure^30^ and found to parallel transfection efficiencies^18^. Conformational analysis by CD showed that MTP_KH_ was α-helical when bound to pDNA (N/P 0.5). This structural motif is not only essential to the function of mitochondria-targeting peptides^18^,^31^, but also allowed topological changes to occur in DNA before charge neutralization point^30^.

Collectively, our experimental data indicate that MTP_KH_, even before charge neutralization point (N/P 0.5), could bind/condense pDNA into a size (approximately 350 nm) and structure suited for the endocytic mechanism^23^,^24^, and takes a predominant form (α-helical structure) favourable for both cellular^32^ as well as mitochondrial import^31^. Although, at present, we are unsure of the exact delivery mechanism into the mitochondria by our peptide-based vector, it is possible that the mitochondrial-targeted complex dissociates upon contact with the surface of the mitochondria^33^ and the pDNA is imported via the voltage-dependent anion channel followed by the adenine nucleotide translocator^9^. Intracellular penetration was not hindered by the negative surface charge of the complex (approximately −25 mV), which was also the case for other anionic peptide^15^ or peptide-pDNA complex^17^, suggesting that electrostatic interaction with the cell membrane is not a critical factor for complex internalization.

We then examined the effect of adding cell-penetrating components (CPP or CPP_KH_) to the MTP_KH_-pDNA complex. Of note was that CPP and CPP_KH_ share the common ability to decrease the size of MTP_KH_-pDNA complexes when employed at N/P 0.5 and higher ratios. Modifications in particle size may very well influence gene delivery efficiency as the mechanism of internalization and intracellular routing are known to be size-dependent^24^. CPP formed only moderately stable particles (15.3 mV) even at the highest N/P ratio while CPP_KH_ formed highly stable particles from N/P 5 to N/P 100 (46.1–57.0 mV) due to an excess of polycations. As ζ-potential is also a measure of colloidal stability^23^, particles with strong positive surface charges (i.e. highly stable complexes) will not dissociate easily to release pDNA in vivo, rendering them inefficient gene transfer vehicles.

Although comparable in most aspects (pDNA binding/compacting properties, size and structural propensity), addition of polycations to CPP undeniably altered its properties, justifying the slight discrepancies in performance between CPP and CPP_KH_. As mentioned previously, helical conformation of cell-penetrating peptides contribute greatly to their internalization^32^, however, the composition and length of these peptides, as well as the number of cationic residues and even specific positioning of residues in the peptide sequence are equally important factors governing the efficiency of their function^19^. Meanwhile, the lesser amount of either CPP or CPP_KH_ (N/P 0.1) failed to improve translocation of more MTP_KH_-pDNA complexes across the cellular membrane whereas higher concentrations of these peptides (N/P 1 to 100) promoted complex stability to a degree that prevented in vivo dissociation, more so in the case of CPP_KH_ with attached polycations.

Overall, the introduction of CPP at N/P 0.5 to preformed MTP_KH_-pDNA complexes, decreased particle sizes and reduced negative surface charges which ultimately led to a significant boost in transfection levels (a maximum of 1.5-fold increase). The net negative particle surface charge appears to be a persistent feature of our formed complexes, confirming that electrostatic interaction with the cell membrane is indeed not crucial for complex internalization. The negative surface charge may have also contributed positively towards the passage of complexes through the cell wall. Positively-charged complexes could be trapped within the cell wall through the formation of new hydrogen bonds with negatively-charged constituent molecules of the cell wall, thereby reducing the number of complexes that are able to reach the cell membrane.

In this study, we introduced a new and feasible strategy for delivery of genes to the mitochondria of *A. thaliana* which involves a simple combination of cell-penetrating peptide and mitochondria-targeting peptide. A low concentration of each peptide component was sufficient to constitute a carrier that enabled pDNA to be delivered into the cells and targeted to the mitochondria, where the imported pDNA was expressed to detectable levels within a short period of time. Characterization of mitochondria-targeting peptide and cell-penetrating/mitochondria-targeting peptide mediated gene transfer revealed that mitochondrial-targeted pDNA delivery is a function of complexation and induction of cellular uptake but not of electrostatically-driven cell membrane association. While the present study has laid the groundwork for rational design of peptide-based gene carriers, further exploration of peptide sequences are instrumental to improve the success rates of cellular/mitochondrial translocation. As we have demonstrated transfection of intact plant mitochondria, we now look forward to the development of highly competent selection/screening strategies towards the creation of a new and powerful genetic engineering technology that can potentially redefine the boundaries of biological research.

## Methods

### Peptides

Mitochondria-targeting peptides [MTP, 1525.88 Da; MTP_KH_, 3913.71 Da] and cell-penetrating peptides [CPP, 1421.88 Da; CPP_KH_, 3809.71 Da] were synthesized by the Research Resources Center of RIKEN Brain Science Institute.

### Plasmid DNA

Plasmids pDONR-cox2:rluc and pDONR-cox2:gfp which contain genes encoding RLuc and GFP, respectively, were constructed according to standard molecular biology protocols^34^. Yeast *cox2* promoter for RLuc gene expression was amplified with Sccox2pFattb1 (5′-GGGGACAAGTTTGTACAAAAAAGCAGGCTTCTCACATCTCCTTCGGCCGGAC-3′) and Sccox2pRrluc2 (5′-ATCATAAACTTTCGAAGTCATTGTTAATTGTAATCTTAATAAATC-3′). RLuc gene was amplified with RlucF2 (5′-ATGACTTCGAAAGTTTATGATC-3′) and RlucR2attb2 (5′-GGGGACCACTTTGTACAAGAAAGCTGGGTTTTATTGTTCATTTTTGAGAAC-3′). The resultant fragments were annealed following a denaturation step and the fused PCR products, designated cox2:rluc, served as a template for amplification with Sccox2pFattb1 and RlucR2attb2. In a similar manner, yeast *cox2* promoter for GFP expression was amplified with Sccox2pFattb1 and cox2pRGFPS65T (5′-CTCCTCGCCCTTGCTCACCATTGTTAATTGTAATCTTAATAAATC-3′). GFP s65t was amplified with GFPs65tF (5′-ATGGTGAGCAAGGGCGAGGAG-3′) and GFPs65tRattb2 (5′-GGGGACCACTTTGTACAAGAAAGCTGGGTTTTACTTGTACAGCTCGTCCATG-3′). The resultant fragments were annealed following a denaturation step and the fused PCR products, designated cox2:gfp, served as a template for amplification with Sccox2pFattb1 and GFPs65tRattb2. All PCR reactions were performed using KOD -Plus- Ver.2 (TOYOBO CO., LTD., Osaka, Japan). The amplified cox2:rluc and cox2:gfp fragments were subsequently cloned into pDONR207 by BP reaction (Life technology Corp., Carlsbad, CA), resulting in pDONR-cox2p:rluc and pDONR-cox2p:gfp, respectively.

### Complex formation

MTP- or MTP_KH_-pDNA complexes were prepared by adding 2.5 *μ*L of 1 mg/mL pDNA to increasing volumes of each mitochondria-targeting peptide at various N/P ratios (0.1, 0.5, 1, 2, 5, 10, 20, 50, 100) and autoclaved Milli-Q water to obtain a final volume of 100 *μ*L. The solution was thoroughly mixed (by repeated pipetting) and allowed to stabilize for 30 min at 25°C. CPP- or CPP_KH_-MTP_KH_-pDNA complexes were prepared in a similar manner, by adding increasing volumes of each cell-penetrating peptide at various N/P ratios (0.1, 0.5, 1, 2, 5, 10, 20, 50, 100) to preformed MTP_KH_-pDNA complexes (N/P 0.5) and autoclaved Milli-Q water to obtain a final volume of 100 *μ*L. The solution was thoroughly mixed and allowed to stabilize for 30 min at 25°C.

### Electrophoretic mobility shift assay, dynamic light scattering and scanning electron microscopy

Assessment of complex stability by electrophoretic mobility shift assay, as well as measurements of particle size and ζ-potential by DLS, were as described previously^17^. Complex size and morphology were also investigated by SEM. Samples were mounted on an aluminium stub, sputter coated with gold and examined in the SEM (JSM6330F, JEOL Ltd., Tokyo, Japan) at an accelerating voltage of 5 kV.

### Ethidium bromide displacement assay

The degree of pDNA binding/condensation by each peptide was determined by EtBr displacement assay. Before measurement, pDNA (78.8 *μ*g/mL) was incubated with EtBr (19.7 *μ*g/mL) for 1 hour. Each peptide was then added to the pDNA-EtBr solution (200 *μ*L total volume). The emission spectra were recorded from 560 to 700 nm with excitation at 526 nm using Spectra MAX M3 (Molecular Devices Corporation, Sunnyvale, CA). Fluorescence intensity was determined after subtracting the background fluorescence of EtBr in the absence of pDNA.

### Circular dichroism spectroscopy

The structure of peptides and conformational changes caused by interaction with pDNA were analysed by CD spectroscopy. Peptides (25 *μ*M) and their complexes with pDNA (N/P 0.5) were stabilized by the addition of 40% (v/v) 2,2,2-trifluoroethanol (TFE), and the CD spectra were acquired using a Jasco J-820 CD spectropolarimeter. Background scans were obtained for 40% TFE in water. Measurements were made using a quartz cuvette with 0.1 cm pathlength. Each spectra represents the average of three scans from 190 to 250 nm with 0.2 nm resolution. The scans were obtained at 20 nm min^−1^ with a bandwidth of 1.0 nm and assignments of secondary structures were based on the method of Yang et al^27^.

### Plant growth conditions and transfection with peptide-pDNA complex

*Arabidopsis thaliana*, which serves as a model plant system in this study, was grown under the same conditions used previously^17^. Leaves were infiltrated with complexes as described^17^, and sampled at intermittent time points of 3 h, 6 h, 12 h, 24 h, 36 h and 48 h.

### *Renilla* luciferase assay

Expression of RLuc gene in the mitochondria was evaluated quantitatively by RLuc assay as detailed here^17^.

### Confocal laser scanning microscopy and Western blot analysis

Expression of GFP gene in the mitochondria was evaluated qualitatively by confocal laser scanning microscopy as detailed here^17^. Prior to microscopic observation, mitochondria were stained with MitoTracker Red CMXRos (Molecular Probes, The Netherlands) as follows: leaves were incubated in a 1 *μ*M dye solution containing phosphate buffered saline (PBS) for 30 min and then washed three times in dye-free PBS to eliminate excess dye. Additionally, GFP expression was detected by Western blot analysis using a suspension of crude mitochondria isolated from leaves previously infiltrated with peptide-pDNA complexes (final pDNA amount of 10 *μ*g). *A. thaliana* leaf mitochondria was prepared according to an established protocol^35^ with these modifications: the starting material consisted of 20–30 leaves; 10 mL of grinding buffer A was used; and following the final step, the crude mitochondria suspension was further concentrated by centrifugation and resuspension to an approximate final volume of 50 *μ*L in wash buffer A. The resultant solution was then separated on a gradient SDS-PAGE gel and transferred to an Invitrolon™ PVDF membrane (Invitrogen, Carlsbad, CA) using a Mini Trans-Blot® SD Semi-Dry Electrophoretic Transfer Cell (Bio-Rad, Hercules, CA). GFP was detected using a mouse monoclonal anti-GFP antibody (1:200; ab38689, Abcam) and goat anti-mouse IgG conjugated with alkaline phosphatase as a secondary antibody (1:2000; sc-2008, Santa Cruz Biotechnology).

### Statistical analysis

SPSS 17.0 for Windows (IBM, Armonk, NY) was employed for statistical analysis. Tukey's Honestly Significant Difference (HSD) test was used in conjunction with analysis of variance (ANOVA) for single-step multiple comparisons. Differences between two means were considered statistically significant at *P* < 0.05 and indicated with asterisks (*). Data in experiments are expressed as means ± standard deviation (*n* = 3) for size and ζ-potential measurements, and (*n* = 4) for transfection efficiencies quantified by RLuc assay.

## Author Contributions

J.C. and K.N. conceived the study and designed the experiments. T.Y. and Y.K. constructed the plasmids. J.C. performed experiments and analysed the data. J.C. wrote and K.N. edited the manuscript.

## Supplementary Material

Supplementary Information Supplementary info
