# Supplementary material for: Gene introduction into the mitochondria of Arabidopsis thaliana via peptide-based carriers
Source: Sci Rep. 2015 Jan 13;5:7751. doi: 10.1038/srep07751 (PMC4291575; doi:10.1038/srep07751)
Supplement: Supplementary Information — Supplementary info [file srep07751-s1.pdf]

Gene introduction into the mitochondria of *Arabidopsis thaliana* via peptide-based carriers

Jo-Ann Chuah[1], Takeshi Yoshizumi[2], Yutaka Kodama[3] & Keiji Numata[1,*]

[1]Enzyme Research Team, Biomass Engineering Program Cooperation Division, Center for Sustainable Resource Science, RIKEN, 2-1 Hirosawa, Wako-shi, Saitama 351-0198, Japan

[2]Institute for Advanced Biosciences, Keio University, 403-1 Nipponkoku, Daihoji, Tsuruoka, Yamagata 997-0017, Japan

[3]Center for Bioscience Research and Education, Utsunomiya University, Tochigi 321-8505, Japan

* Correspondence should be addressed to K.N. (keiji.numata@riken.jp).

Contents

Supplementary Figure 1 Efficiency of pDNA delivery mediated by individual components of the designed peptide-based carrier.

Supplementary Figure 2 Observation of CPP-MTP_{KH}-pDNA complexes by scanning electron microscopy.

Supplementary Figure 3 Cytosolic expression of RLuc gene under the control of different promoters.

Supplementary Table 1 Particle size and polydispersity index (PDI) of particles formed by complexation of pDNA with MTP_{KH} (N/P 0.5) and CPP.

Supplementary Figure 1 Efficiency of pDNA delivery mediated by individual components of the designed peptide-based carrier.

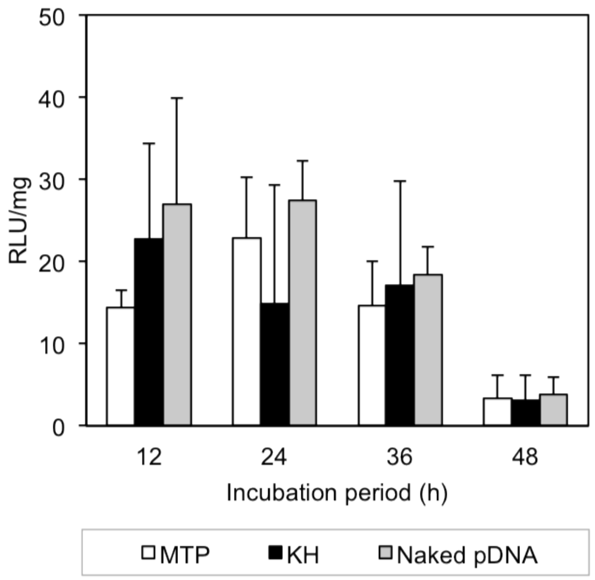

MTP or lysine-histidine copolymer (KH) were used for complexation with pDNA before infiltration into the leaves of *A. thaliana* and incubated for various time periods (12 h, 24 h, 36 h and 48 h) prior to evaluation by RLuc assay. Naked pDNA, treated in the same manner as the peptide-pDNA complexes, served as negative control. Error bars represent standard deviations (n = 4). Complexes formed using MTP or lysine-histidine copolymer (N/P 0.5) or naked pDNA, when delivered to leaves, mediated negligible levels of expression.

Supplementary Figure 2 Observation of CPP-MTP_{KH}-pDNA complexes by scanning electron microscopy.

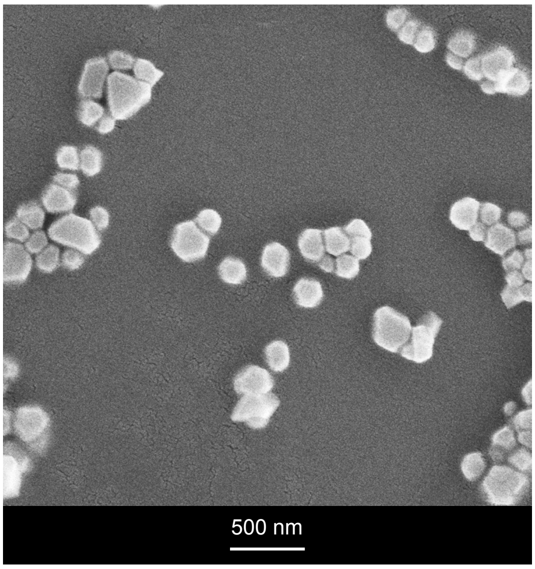

Complexes formed by sequential addition of peptides to pDNA (MTP_{KH} at N/P 0.5 followed by CPP at N/P 0.5) were relatively uniform in size (approximately 200 nm).

Supplementary Figure 3 Cytosolic expression of RLuc gene under the control of different promoters.

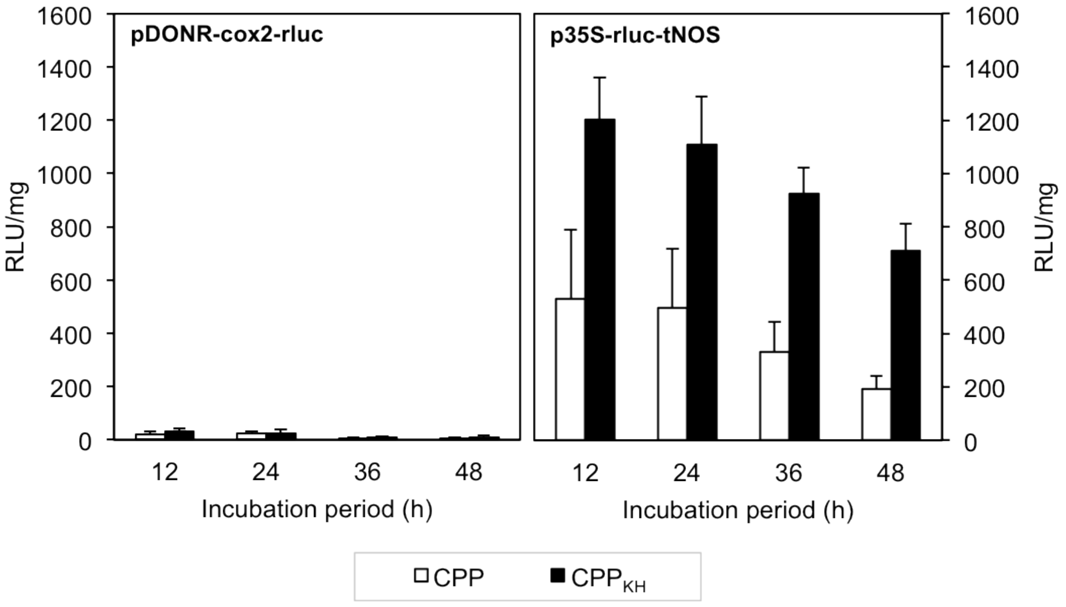

Plasmid constructs containing the RLuc gene expressed by a mitochondrial promoter (pDONR-cox2-rluc) or cytosolic promoter (p35S-rluc-tNOS) were delivered into the cell cytoplasm using either CPP or CPP$_{KH}$. Evaluation by RLuc assay, following various incubation periods (12 h, 24 h, 36 h and 48 h), showed that the mitochondrial-specific cox2 promoter mediates only negligible levels of RLuc gene expression in the cytoplasm. Functionality of CPP and CPP$_{KH}$ were confirmed by the noticeably higher levels of RLuc gene expression driven by the constitutive p35S promoter. Error bars represent standard deviations (*n* = 4).

Supplementary Table 1 Particle size and polydispersity index (PDI) of particles formed by complexation of pDNA with MTP_{KH} (N/P 0.5) and CPP.

N/P ratio	Average mean hydrodynamic diameter (nm)	PDI
50	2160	0.7
100	1620	0.7

N/P is defined as the number of amine groups from the peptide divided by the number of phosphate groups from pDNA. The addition of higher concentrations of CPP (N/P 50 or N/P 100) caused aggregates to form, resulting in formulations with large variability in size (based on the high PDI values).
